# Stakeholders’ Perceptions of the Nature-Based Healing Industry in South Korea: A Q Methodology Study

**DOI:** 10.3390/healthcare13161990

**Published:** 2025-08-14

**Authors:** Moon Hee Yu, Ji Seong Yi, Seo Jung Shin, Jae Soo Kim, Jeong Hyun Kim, Yu Cheon Kim, Song Yi Lee

**Affiliations:** 1 Counselling and Coaching Department, Graduate School, Dongguk University-Seoul, Seoul 04620, Republic of Korea; thebraintoolbox@gmail.com (M.H.Y.); 2020126709@dgu.ac.kr (J.S.Y.); tjwjd620822@gmail.com (S.J.S.); 2 Department of Food Industry Management, Dongguk University-Seoul, Seoul 04620, Republic of Korea; jsoobest@dongguk.edu; 3 Rosen College of Hospitality Management, University of Central Florida, Orlando, FL 32827, USA; jeonghyun.kim@ucf.edu

**Keywords:** healing industry, Q methodology, public choice theory, subjective perception, nature-based therapy, policy design, Republic of Korea

## Abstract

**Background/Objectives:** This study explores the subjective perceptions of stakeholders in South Korea’s nature-based healing industry and employs Q methodology to classify their viewpoints. As the healing industry continues to evolve across sectors such as forest therapy, marine healing, and healing agriculture, understanding diverse stakeholder perspectives is essential for informing coherent and inclusive policy development. **Methods:** A total of 25 participants—including policymakers, practitioners, and service users—sorted 39 statements derived from academic and media sources. This study analysed the data using Ken-Q software, applying principal component analysis with Varimax rotation. **Results:** The results revealed four distinct perception types: (1) a comprehensive and service-oriented type emphasising universal access and public benefit, (2) a professionalism-oriented type advocating for systematic administration and regional development, (3) a differentiation-oriented type concerned with conceptual clarity and distinctiveness, and (4) a sustainability-oriented type emphasising long-term impacts and collaborative structures. **Conclusions:** These findings highlight the multi-dimensional nature of stakeholder perceptions and suggest the need for differentiated governance strategies. By incorporating public choice theory and complementary insights from health economics, this study provides an empirical foundation for understanding stakeholder-driven policy considerations in developing nature-based healing services.

## 1. Introduction

Modern societies strive to improve the quality of life while addressing physical and mental health challenges. The World Health Organisation [1] emphasises that natural environments and cultural engagement play a vital role in health promotion and psychological well-being, suggesting their integration into national health and welfare policies. In line with this, the nature-based healing industry—a convergence of welfare, agriculture, culture, healthcare, and technology—has gained attention as an emerging sector designed to promote holistic physical and mental health.

In South Korea, increasing public interest in quality of life has led to the growing recognition of “healing” as a new social value that extends beyond physical health to encompass emotional and psychological recovery. In response to this societal demand, the South Korean government has actively promoted the healing industry as a strategy for national and regional development. Governmental agencies such as the Ministry of Agriculture, Food and Rural Affairs, the Korea Forest Service, and the Ministry of Oceans and Fisheries, along with local municipalities, have initiated various programmes and infrastructure projects rooted in rural experiences, forest therapy, and marine healing [2,3,4,5,6]. These efforts support broader policy goals to revitalise local economies by integrating regional resources and improving residents’ quality of life.

Notably, in 2020, the Ministry of Agriculture, Food and Rural Affairs enacted the Act on Healing Agriculture, providing a legal foundation for research and development in the field and supporting programmes that integrate agriculture with healing interventions [7]. Since the enactment of this legislation, public interest in healing agriculture has grown, accompanied by increasing media coverage and expanding academic discourse [8]. Consequently, practitioners and researchers in the healing industry are steadily accumulating field practices and empirical evidence.

Despite this growth, the sustainable development of the healing industry requires institutional support, along with enhanced public understanding and engagement, both of which depend on empirical research. However, despite government leadership, South Korea’s healing industry struggles with fragmented conceptual frameworks and inconsistent policy directions across ministries. These issues continue to confuse service providers, users, and stakeholders [9]. Effectively addressing these structural issues requires a clear understanding of stakeholder perceptional typologies and the frameworks that influence their formation. Particularly, the subjective perceptions of policymakers, practitioners, and service users are critical in advancing a coherent and inclusive healing industry.

Stakeholders’ decisions and behaviours play a crucial role in shaping the effectiveness and public acceptance of services and policies. This influence is especially significant in local or public projects that involve multiple actors with diverse interests, where decision-making processes are often complex and not based solely on rational calculations.

Accordingly, this study draws on four theoretical perspectives: public choice theory, behavioural economics, behavioural public choice theory, and health economics. Traditional public choice theory posits that public actors, like individuals, behave as rational agents seeking to maximise self-interest [10,11]. This approach provides a useful lens for analysing the motivations and strategic behaviours of policy suppliers.

Tullock [12] conceptualised the government as a natural monopoly, asserting that political authority, exercised without competition, mirrors the characteristics of economic monopolies. In such structures, bureaucrats may monopolise information on the quality and quantity of services, impeding accurate performance assessments by politicians and the public [11]. Moreover, policymakers may distort policy outcomes due to cognitive biases and bounded rationality, resulting in decisions driven more by personal or political interests than by public welfare [13].

From this perspective, the government comprises self-interested agents, and effective governance requires alignment between institutional design and performance management systems [14]. Such alignment is crucial for achieving organisational effectiveness and policy objectives [15].

Behavioural economics is a theoretical framework developed to address the limitations of traditional economic models, including public choice theory. By accounting for individuals’ bounded rationality and psychological and cognitive biases, it enhances the realism of decision-making and complements institutional designs grounded in classical public choice theory [16]. Building on this foundation, behavioural public choice theory applies these insights to improve the efficiency of policy decision-making and design, challenging the assumption of purely rational choice by highlighting the role of cognitive limitations, emotions, and contextual factors [17].

Stakeholders may respond to policy options using heuristics—mental shortcuts that can lead to biases such as status quo bias, omission bias, and affective forecasting errors, rather than through economic rationality [18,19]. This behavioural perspective holds particular relevance for the Korean healing industry, where unclear concepts, ambiguous value criteria, and overlapping roles between public and private providers may induce cognitive biases in how stakeholders perceive and evaluate related policies.

In conjunction with these theories, health economics offers a structural and institutional analytical framework for examining the economic attributes and policy implications of the healing industry. As a field concerned with the efficiency, effectiveness, value, and behavioural dimensions of producing and consuming health services, health economics offers key insights into how stakeholder participation, decision-making processes, and socio-political contexts shape health policy [20]. It is inherently interdisciplinary, requiring integration of perspectives from economics, public health, sociology, and political science.

Goeree and Diaby [21] emphasise the value of such cross-disciplinary approaches, particularly in applying economic reasoning to complex clinical and institutional decisions. Sittimart et al. [22] explain that different evaluative perspectives, such as those of society, health systems, payers, or patients, can lead to varying policy outcomes and resource allocation decisions. This plurality of perspectives is particularly pertinent to the healing industry, where overlapping policy goals and beneficiaries complicate standardised economic assessments.

In practical terms, health economics emphasises the distinction between public and private goods, the role of externalities, and the necessity of resource allocation frameworks that ensure cost-effectiveness and equity. For instance, Jin et al. [23] demonstrated how supply chain disruptions during public health crises significantly impacted regional economic stability, highlighting the interdependence of health systems and broader economic structures.

These findings underscore the relevance of evaluating the healing industry’s economic attributes, such as the distinction between public and private goods and the presence of externalities, as well as strengthening the rationale for public investment and developing feasible reimbursement schemes.

Roncarolo et al. [24] identified governance, service delivery, and the allocation of human and infrastructural resources as key factors for ensuring the long-term sustainability of health systems. Sim et al. [25] support this view in their study of stakeholder perceptions regarding the reimbursement of digital therapeutics, a type of non-clinical service. They found that stakeholders held positive attitudes and acknowledged the practical value of digital therapeutics, but differing views on reimbursement mechanisms posed major implementation challenges. They concluded that reducing uncertainty and generating clinical evidence—through tax-based funding, innovation funds, and pilot studies supported by the National Health Insurance—is essential. These findings collectively underscore the need for comprehensive and structured policy design for the stable institutionalisation of non-clinical services such as the healing industry.

The healing industry in South Korea lacks a clear institutional definition or standardised industrial classification. Its multi-stakeholder nature presents a range of perspectives regarding its identity and development trajectory. These varying perceptions critically influence policy acceptance and implementation. Therefore, a systematic exploration of stakeholders’ subjective views is imperative [26].

Previous studies using the Q methodology have analysed stakeholder perceptions in related areas such as ecotourism, heritage-based regeneration, and urban park development. For instance, a Q methodological study on the development of the Maha Ecotourism Site in Pyeongchang identified five perception types and proposed corresponding policy directions [26]. Another study conducted in Seocheon, South Korea, examined how residents, experts, and public officials differed in their perceptions of ecotourism infrastructure [27]. More recently, a Q study explored perceptions of healing food among consumers and professionals, contributing to a more nuanced understanding of the multi-dimensional value of healing-related domains [28].

However, most existing studies focus on specific subdomains or small-scale projects, leaving a gap in comprehensive research on the healing industry as a whole—particularly studies that explore the differing values and perceptions of key actors involved in state-led initiatives at the national or regional policy levels.

To address this gap, the present study employs Q methodology to investigate the subjective perceptions of diverse stakeholders, including government agencies (agriculture, forestry, marine, and local governments), field practitioners, and participants in the healing industry. This study identifies and categorises stakeholder perception types and characteristics, offering foundational insights to inform the conceptual development and future directions of the healing industry.

Accordingly, this study systematically explores the subjective perception types of key stakeholders to enhance the effectiveness and acceptance of healing industry policies implemented at national and regional levels. Through this, this study provides foundational insights for the conceptual development and future direction of the healing industry.

This study employs Q methodology to identify and classify the perception types and characteristics of diverse stakeholders, including government departments (such as agriculture, forestry, oceans, and local governments), field practitioners, and service users.

Through this approach, this study offers practical implications for policy orientation and institutional design in the healing industry.

The research questions (RQ) are as follows:

RQ1. What are the subjective perception types of stakeholders regarding the nature-based healing industry?

RQ2. What are the characteristics of each perception type among stakeholders toward the nature-based healing industry?

## 2. Materials and Methods

### 2.1. Q Methodology Research Procedure

William Stephenson developed the Q methodology in the 1930s as a systematic research approach to explore human subjectivity. In this context, subjectivity refers to expressions of thoughts, feelings, and attitudes that individuals can articulate to themselves or others. Q methodology enables participants to engage actively with a set of statements—called stimuli—and rank-order them, thereby structuring their perspectives within a comparative framework [29,30].

This methodology integrates data collection and analysis into a single, unified process designed to investigate subjectivity empirically. Unlike traditional R methodology, which treats variables as the unit of analysis, Q methodology considers individuals as variables and conducts factor analysis to uncover shared patterns of thought across participants. Data collection involves quantitative and narrative elements, allowing researchers to adopt a mixed-methods approach that structures subjective viewpoints around a conceptual framework [31,32].

Since the 2000s, Q methodology has rapidly gained traction across disciplines such as the social sciences, environmental studies, and healthcare. A recent review by Dieteren et al. [33] confirms its widespread adoption, particularly in fields that deal with complex values and stakeholder interests.

In public administration—traditionally dominated by R methodological approaches such as large-scale surveys, correlation analysis, and regression models—Q methodology provides a complementary lens. Miller and Whicker [34] argue that public administration often involves multiple stakeholders with conflicting value systems and complex interests, making it difficult to capture decision-making dynamics through variable-based analyses alone. They emphasised the necessity of in-depth analysis that probes the subjective beliefs and priorities underpinning public policies and institutions. Building on this perspective, Brown et al. [35] theorised that public issues involve more complexity than simple dichotomies of agreement and disagreement. Rather, Q methodology reveals the multi-layered structure of stakeholders’ value orientations, serving as a tool for visualising divergent viewpoints and facilitating consensus-building in policymaking.

This methodological strength—its ability to capture and structure complex subjective value systems—has made Q methodology particularly useful in areas such as public resource management and environmental policy. For instance, Steelman and Maguire [36] used Q methodology to identify diverse stakeholder values in forest resource management, highlighting its role in conflict resolution and participatory decision-making. Likewise, Dickinson et al. [37] applied the Q methodology to explore stakeholders’ perceptions of joint commissioning in public service design, demonstrating its potential to inform the development of inclusive policy alternatives.

Given these strengths, researchers consider the Q methodology especially well-suited for analysing the distinctive characteristics of South Korea’s nature-based healing industry. It allows them to structure diverse stakeholder perspectives and explore multi-dimensional value interactions within the sector. This approach supports Miller and Whicker’s [34] assertions about the inherent complexity of public administration and offers practical insights into achieving policy consensus and mediating conflicts.

The Institutional Review Board (IRB) of Dongguk University reviewed and approved this study (IRB No. DUIRB-2024-11-08). The Q methodological procedure consists of five steps: (1) developing the Q population, (2) selecting the Q sample, (3) recruiting the P sample (participants), (4) conducting the Q-sorting process, and (5) analysing the data. Table 1 summarises these steps.

#### 2.1.1. Q Concourse Organisation

In Q methodology, the Q concourse refers to a collection of subjectively meaningful expressions that are communicable and form the foundation for scientific inquiry. Wolf [38] emphasised that compiling statements or opinions mechanically, without considering their context, undermines the interpretive framework essential to factor analysis. He argued that a valid concourse must have structural coherence, reflect social and experiential contexts, and offer meaningful interpretive potential.

Unlike purely informational communication, researchers derive the elements of a subjective concourse from sources open to various interpretations, such as academic literature, grey literature, media content, interviews, and online forums [31,33]. Among these, Yoon [39] and Sass [40] developed their Q concourses exclusively from published academic sources. Similarly, Bublitz–Berg et al. [41] built their concourse on employees’ subjective perceptions of organisational leadership by analysing scholarly literature, news articles, and media posts.

The present study constructed the Q concourse by collecting content related to six core keywords representing Korea’s nature-based healing industry: healing industry, forest healing, marine healing, healing agriculture, garden therapy, and healing tourism. Between 11 and 17 November 2024, we conducted a comprehensive search across academic databases, grey literature, and various media platforms. We organised the search results in Microsoft Excel, yielding the following number of items per category: 89 for the healing industry, 128 for forest healing, 35 for garden therapy, 30 each for healing tourism, healing agriculture, and marine healing, and an additional 57 from miscellaneous sources. We collected 399 statements to form the initial Q population.

#### 2.1.2. Q Sample Selection

Researchers draw the Q sample as a subset from the broader Q concourse and construct it to reflect the range and diversity of perspectives on the topic. Researchers develop the Q sample through an iterative process of expert review and refinement to ensure it is comprehensive and representative. The process involves rearticulating raw ideas into clearer, more meaningful, and natural statements [29,31]. While a typical Q sample consists of approximately 30 to 60 statements, the actual number varies depending on the research topic [33,42].

This study began by categorising the initial 399 statements collected from the Q concourse into broad themes such as concepts and purposes, effectiveness and function, convergence, programmes, target groups, and miscellaneous topics. We then further refined these themes into 36 intermediate thematic categories. After merging redundant or overlapping content, we edited the statements to ensure thematic representativeness and semantic clarity, ultimately identifying 57 preliminary Q sample statements.

Next, three researchers with prior experience in Q methodological studies reviewed the 57 statements. Through consensus-building discussions, they resolved discrepancies and conducted an initial content validity check, reducing the number of statements to 41. Two university professors with extensive experience in Q methodology instruction and research then performed a second round of content validation. Based on their expert evaluations, the researchers finalised a set of 39 Q sample statements.

#### 2.1.3. P Sample Organisation

Q methodology defines the P sample as the group of individuals who evaluate the Q sample by performing a procedure known as Q sorting. Researchers purposefully select individuals for the P sample to capture a wide range of subjective viewpoints relevant to the research topic. Although many scholars recommend including 20 and 60 participants, this range does not aim to support statistical generalisation. Instead, Q methodology primarily seeks to uncover distinct patterns of subjectivity embedded within the sample [29,42].

Reflecting this methodological orientation, Q studies typically do not require large P-samples. Rather, they aim to capture the rich diversity of subjective perspectives through carefully selected smaller groups. A systematic literature review by Dieteren et al. [33] found that 21% of Q studies deemed small samples sufficient, and the majority (64.4%) included between 20 and 50 participants. In line with this standard, we conducted the present study with 25 participants, which is consistent with the typical sample size in Q methodological research.

Accordingly, we employed purposive sampling to ensure the inclusion of diverse stakeholder voices across five core sectors of South Korea’s nature-based healing industry: forest healing, marine healing, healing agriculture, garden therapy, and healing tourism. We recruited 25 participants—including policymakers, field practitioners, and service users—between 11 and 14 November 2024. We designed the sampling to reflect the diversity of perspectives shaping the field, rather than to produce statistically generalisable results.

#### 2.1.4. Q Sorting

Q sorting is the central technique of Q methodology, in which participants (the P sample) actively rank-order a set of Q-sample statements based on their subjective perspectives [42,43]. While traditional psychology often explores correlations between variables, Q methodology emphasises the structure of individuals’ thoughts, attitudes, and emotions. Stephenson [30] posited that the ranking process reflects varying levels of psychological significance and uncovers the structural meaning embedded in an individual’s viewpoint. Accordingly, Q sorting offers a scientific model of personal subjectivity [44,45].

**Figure 1:**
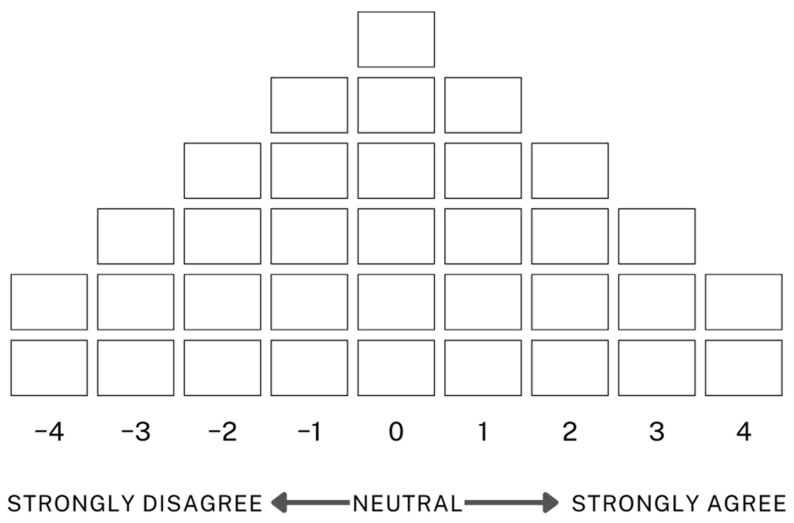
Q sorting distribution chart.

Researchers can implement Q sorting in various formats, including forced distribution or free sorting. In particular, forced distribution requires participants to rank each statement in relation to others rather than evaluating them in isolation, thereby revealing a coherent internal meaning system. This study employed a forced distribution method using a quasi-normal distribution, as illustrated in Figure 1. We instructed participants to sort the 39 Q sample statements across a nine-point scale, assigning a fixed number of items to each level of agreement or disagreement [42].

The wide geographic scope of Korea’s nature-based healing industry, including forests, farms, and marine areas, required participants from across the country. Therefore, we conducted Q sorting using an online platform (www.qmethodsoftware.com (accessed on 26 November 2024)). Before beginning the sorting task, participants received detailed email instructions on how to access and use the platform. The platform’s embedded instructions provided additional guidance to assist participants through each step of the sorting process. The Q sorting process took place between 29 November and 3 December 2024, allowing participants to complete the task at their convenience.

Although 26 individuals initially participated, one did not complete the process. Ultimately, this study obtained valid Q-sorting data from 25 participants.

The final P sample included nine males and sixteen females, with overlapping roles and experiences across multiple sectors of the healing industry: marine healing (*n* = 9), forest healing (*n* = 7), healing tourism (*n* = 7), healing agriculture (*n* = 5), and garden therapy (*n* = 4). Their professional roles also overlapped, including policy-related work (*n* = 10), planning and field operations (*n* = 12), external institutional collaboration (*n* = 8), programme facilitation (*n* = 7), domain expertise (*n* = 6), and others (*n* = 2). Six service users also participated. In terms of experience, six participants had over 11 years of experience in the healing industry, six had 6–10 years, four had less than 1 year, and two had 1–3 years.

#### 2.1.5. Data Analysis

The research team collected Q-sorting data from 25 participants through an online platform and converted the responses into an Excel spreadsheet for analysis using Ken-Q (v1.3.1) software. Q methodology builds on factor analysis and follows a three-step analytical process. Although this structure resembles that of R methodology, Q methodology differs in its unit of analysis: R methodology analyses variables, whereas Q methodology focuses on individuals to reveal the subjective structure of participants’ perceptions [45].

Typically, researchers begin the analysis by computing correlation matrices across all participants’ Q sorts. Next, they extract common patterns using factor analysis, often employing methods such as principal component analysis (PCA) or common factor analysis. Finally, they rotate the extracted factors to improve interpretability, using an objective method like Varimax or a subjective, judgmental approach [31,43].

In this study, we used PCA to extract factors. PCA identifies components in order of the variance they explain, beginning with the one that accounts for the greatest variance. After extraction, we applied the Varimax method—the most commonly used objective rotation technique in Q methodology—to clarify the factor structure.

We used Ken-Q (v1.3.1) to calculate the relative significance of each factor using eigenvalues and generated correlation coefficients among factors and z-scores for each statement within each factor. We interpreted the factor analysis results using several key metrics: eigenvalues, percentage of explained variance, cumulative variance, and z-scores. The z-scores indicated the relative importance of each statement within a given factor and helped identify the defining characteristics of each perception type.

## 3. Results

### 3.1. Analysis Results

This study set out to identify and explore the subjective perception of stakeholders involved in South Korea’s nature-based healing industry. Using Q methodology, we analysed the Q sorting data and extracted four distinct types. We retained factors based on the statistical criterion of eigenvalues greater than 1.0. Since the percentage of explained variance beyond the fourth factor showed no meaningful incremental increase, we determined that additional factors offered limited analytical value.

The analysis confirmed that the four identified factors provided the most coherent and comprehensive structure of stakeholder perceptions, collectively explaining 63% of the total variance. Following practical guidelines that recommend retaining factors with eigenvalues greater than 1.0 [31], we selected this four-factor solution as it effectively captures the main viewpoints on the topic. Table 2 presents the eigenvalues and the percentage of variance explained by each factor.

Table 3 presents the inter-factor correlation matrix derived from the factor analysis of the Q-sort data. The inter-factor correlations, calculated after varimax and manual rotations, ranged from 0.1912 to 0.5651. Type 1 correlated positively with Types 2 and 4 but showed a weak negative correlation with Type 3. Type 3 also exhibited a weak negative correlation with Type 4. Meanwhile, the correlation between Types 2 and 4 was minimal, indicating limited similarity between these two perspectives. These patterns underscore the distinctiveness of the four extracted perception types and confirm their differentiation based on subjective viewpoints.

Table 4 presents the composition of the P sample and their corresponding factor loadings for each type, derived from the factor analysis of the Q-sort data. We calculated the factor loadings using principal component analysis (PCA) for factor extraction, followed by varimax and manual rotations, representing each participant’s alignment with the identified subjective viewpoints. Within each type, the participant with the highest factor loading best represents the corresponding perspective. Types 1 and 2 each included eight participants, while Types 3 and 4 comprised three and six participants, respectively. Participants P3, P6, P1, and P9 exhibited the highest factor loadings for Types 1 through 4, respectively, making them the most representative individuals for each perception type.

### 3.2. Perception Type Characteristics

To identify stakeholders’ perceptions of the Korean nature-based healing industry, this study applied Q methodology. Supplementary S1 presents the factor arrays for the 39 Q statements, derived from the factor analysis described earlier. The arrays include the z-scores and Q-sort values, reflecting each statement’s position across the four perception types.

Three interpretive criteria guided the identification of key distinguishing statements. First, we prioritized Q statements with the highest absolute z-scores (≥|1.0|), as these reflect the items each type agreed or disagreed with most strongly. Second, we considered Q-sort values that revealed the most distinctive differences between types; Supplementary S1 presents these items, marked with an asterisk (*). Third, we incorporated participants’ qualitative comments explaining the two statements with which they most strongly agreed and disagreed during the Q sorting process.

Using this analytical framework, we identified and interpreted four distinct perception types, as follows:

- Type 1: A comprehensive and service-oriented type emphasising universal access and public benefit
- Type 2: A professionalism-oriented type advocating for systematic administration and regional development
- Type 3: A differentiation-oriented type questioning conceptual clarity and distinctiveness
- Type 4: A sustainability-oriented type highlighting long-term effects and collaborative structures

#### 3.2.1. Type 1: A Comprehensive and Service-Oriented Type Emphasising Universal Access and Public Benefit

Type 1 reflects a clear policy-oriented perspective that emphasises providing healing services universally, including to individuals with medical conditions, while asserting confidence in the distinctiveness of healing programmes. Type 1 included eight participants. Among them, seven worked in policy, planning, or operational aspects of the healing industry, and five had more than six years of relevant work experience.

The statements with which Type 1 most strongly agreed included the following:

- Healing services should be available to the entire population (Q8, Z = 1.672)
- The healing industry should address physical and mental recovery in a balanced way (Q1, Z = 1.589)
- Scientifically and medically proving the effectiveness of the healing industry can help with its promotion (Q14, Z = 1.492)
- Cross-sectoral cooperation between policy, health, psychology, and medical fields is necessary to advance the healing industry (Q17, Z = 1.439)
- The healing industry must encompass social, cultural, and educational aspects of health (Q2, Z = 1.313).

In contrast, Type 1 participants strongly disagreed with statements such as the following:

- The healing industry should exclude individuals with medical conditions requiring treatment (Q9, Z = −1.780)
- The intended users of the healing industry remain unclear (Q11, Z = −1.619)
- Healing programmes often feel indistinguishable from general wellness tourism (Q16, Z = −1.516)
- In some cases, the development of healing infrastructure and commercial interests ends up damaging nature (Q34, Z = −1.474)
- Researchers have not clearly defined how they are measuring the outcomes of the healing industry (Q38, Z = −1.313).

Type 1 participants’ subjective comments illustrate a shared view of the healing industry as a universal public service grounded in accessibility, legitimacy, and developmental potential.

P3, with over 11 years of experience in policy and inter-organisational collaboration, emphasised that “All citizens should be the target of the healing industry” and that healing must integrate physical and mental aspects to be meaningful—reflecting a belief in its essential public function.

P15 described healing as rooted in a fundamental human instinct to reconnect with nature, suggesting an intuitive yet theoretically grounded understanding of its therapeutic potential. Complementing these views, P21 framed the healing industry as a “universal welfare tool” and noted the increasing institutionalisation of healing programmes, pointing to Korea’s growing global competitiveness.

Together, these statements indicate that participants perceive the healing industry as requiring a structured system of governance and professional standards to function effectively as a regional industry and response to rural decline.

Compared to the other types, Type 1 showed uniquely strong agreement with the following statements:

- Healing services should be available to the entire population (Q8, Z = 1.672)
- The healing industry should address physical and mental recovery in a balanced way (Q1, Z = 1.589)
- The healing industry must encompass social, cultural, and educational aspects of health (Q2, Z = 1.313).

However, Type 1 showed the strongest disagreement with the following statement:

- Researchers have not clearly defined how they are measuring the outcomes of the healing industry (Q38, Z = −1.313).

Based on these findings, we defined Type 1 as a perspective that views the healing industry as a universal welfare system. Stakeholders with this view emphasise accessibility for all and broad societal benefit. This type highlights the industry’s envisioned positive role in society, extending beyond any single functional domain.

Therefore, we labelled Type 1 the “Comprehensive and Service-Oriented Type”.

#### 3.2.2. Type 2: A Professionalism-Oriented Type Advocating for Systematic Administration and Regional Development

Type 2 reflects the view that the healing industry must adopt a structured system of operation and management to develop as a regional industry and function as a strategic response to rural decline. We identified eight participants in this group. Among them, five worked in policy, planning, or field-level implementation, and four had over six years of relevant work experience.

The statements with which Type 2 participants strongly agreed included the following:

- The healing industry needs a government body dedicated to its oversight (Q18, Z = 1.893)
- The healing industry needs clear and systematic guidelines for its operation and management (Q22, Z = 1.798)
- The healing industry can serve as a future-oriented strategy to address rural depopulation (Q19, Z = 1.795)
- Collaboration with experts in the planning and operation phases is essential for administrative practitioners (Q24, Z = 1.298)
- The healing industry should pursue long-term effects through repeated and continuous experiences rather than one-time programmes (Q33, Z = 1.292)
- A business structure that ensures income for residents is crucial for the sustainability of the healing industry (Q20, Z = 1.190).

On the other hand, the statements with which Type 2 strongly disagreed included the following:

- Formal facility standards overly constrain the healing industry (Q26, Z = −1.552)
- Healing programmes are more effective for physical health than for mental health (Q4, Z = −1.411)
- The healing industry should exclude individuals with medical conditions requiring treatment (Q9, Z = −1.399)
- In some cases, the development of healing infrastructure and commercial interests ends up damaging nature (Q34, Z = −1.381).

Type 2 participants expressed concern about the healing industry’s lack of clear standards and a defined professional identity.

P6 warned that “When healing programmes are implemented indiscriminately without clear standards, it causes confusion among users”, and emphasised their potential to attract visitors and industry workers as a strategy for rural revitalisation.

P2 shared a similar view, noting that “Due to a lack of professionalism, the healing industry sometimes feels like just a basic wellness programme”, pointing to insufficient differentiation from existing services.

P17 added that “customized strategies reflecting regional characteristics” are essential for sustainable local development.

Together, these statements suggest that participants viewed the healing industry as requiring structured governance and localized adaptation—combining formal system-building with regional responsiveness.

Compared to other types, Type 2 expressed the strongest agreement with the following statements:

- The healing industry needs a government body dedicated to its oversight (Q18, Z = 1.893)
- The healing industry can serve as a future-oriented strategy to address rural depopulation (Q19, Z = 1.795).

These results indicate that Type 2 places particular emphasis on industrial structure, systematic administration, and the need for formal governance.

Therefore, we labelled Type 2 as the “Professionalism-Oriented Type”.

#### 3.2.3. Type 3: A Differentiation-Oriented Type Questioning Conceptual Clarity and Distinctiveness

Type 3 participants believe that the healing industry lacks clear conceptual and terminological definitions and fails to distinguish itself from other services. This group emphasises the need for stronger policy direction and definitional clarity. The three participants in this group were service users within the healing industry.

Statements with which Type 3 strongly agreed included the following:

- Healing programmes often feel indistinguishable from general wellness tourism (Q16, Z = 1.700)
- Indoor healing programmes often lack differentiation from those offered in urban settings (Q37, Z = 1.207)
- The healing industry needs clear and systematic guidelines for its operation and management (Q22, Z = 1.277)
- The effects of healing programmes often fade quickly after the experience ends (Q36, Z = 1.700)
- Due to a lack of public promotion, general awareness of healing programmes remains low (Q23, Z = 1.193).

Conversely, this group strongly disagreed with the following statements:

- Korea’s healing industry has developed to the point of attracting global attention (Q29, Z = −2.225)
- Although healing programmes can be expensive, they are worth the cost (Q30, Z = −1.999)
- The perception of the healing industry is as a form of welfare that supports vulnerable or marginalized groups (Q10, Z = −1.243)
- The healing industry contributes to raising environmental awareness (Q35, Z = −1.751)
- The healing industry helps form social bonds by providing opportunities for interaction (Q7, Z = −1.396).

Type 3 participants consistently expressed frustration with the lack of clarity and distinction in the healing industry.

P1 remarked, “The public still does not seem to understand fully what the healing industry is. It would not feel strange to call it simply wellness tourism”, highlighting a blurred boundary between healing and commercialized services.

P11 reinforced this perception, stating, “If a service cannot prove its worth, I would not go out of my way to experience it”, and added, “I did not feel that there was enough uniqueness or rarity to justify traveling long distances”.

P22 further emphasised that “the concepts and terminology of the healing industry remain vague. Its direction, scope, access points, and target users are unclear”.

Together, these statements point to a demand for conceptual refinement, definitional coherence, and policy-level clarification, as users remain unconvinced of the industry’s value and distinctiveness.

Compared to other types, Type 3 particularly strongly agreed with the following statements:

- Healing programmes often feel indistinguishable from general wellness tourism (Q16, Z = 1.700)
- Indoor healing programmes often lack differentiation from those offered in urban settings (Q37, Z = 1.207)
- The effects of healing programmes often fade quickly after the experience ends (Q36, Z = 1.700).

In contrast, Type 3 strongly disagreed with the following:

- Although healing programmes can be expensive, they are worth the cost (Q30, Z = −1.999), and
- The healing industry helps form social bonds by providing opportunities for interaction (Q7, Z = −1.396).

These results suggest that Type 3 emphasises the healing industry’s lack of conceptual clarity and consistent policy direction, calling for a more precise definition and clearer strategic distinction from related sectors.

Accordingly, we labelled Type 3 as the “Differentiation-Oriented Type”.

#### 3.2.4. Type 4: A Sustainability-Oriented Type Highlighting Long-Term Effects and Collaborative Structures

Type 4 participants emphasise the importance of achieving long-term effects through differentiated programmes and including a broad range of participants. Six participants were in this group, three with over 11 years of experience in the healing industry and three with less than three years.

Statements with which Type 4 strongly agreed included the following:

- The healing industry should pursue long-term effects through repeated and continuous experiences rather than one-time programmes (Q33, Z = 1.678)
- Collaboration with experts in the planning and operation phases is essential for administrative practitioners (Q24, Z = 1.598)
- Although healing programmes can be expensive, they are worth the cost (Q30, Z = 1.492)
- The healing industry requires a redefinition of concepts and terminology, with clear boundaries (Q15, Z = 1.393).

In contrast, Type 4 participants strongly disagreed with the following:

- The healing industry should exclude individuals with medical conditions requiring treatment (Q9, Z = −2.273)
- Healing programmes often feel indistinguishable from general wellness tourism (Q16, Z = −1.777)
- The effects of healing programmes often fade quickly after the experience ends (Q36, Z = −1.507)
- Indoor healing programmes often lack differentiation from those offered in urban settings” (Q37, Z = −1.337).

Type 4 participants emphasise sustainability grounded in repeated, meaningful experiences and collaborative structures.

P9 stated, “It is essential to build local ecosystems in close connection with the community and local providers to ensure sustainability”, reflecting a view of healing as an embedded, place-based system.

P12 echoed this orientation, noting that “Clear principles and guiding propositions are necessary for sustainable development”, suggesting the need for conceptual coherence alongside structural integration.

P18 stressed that “The essence of healing lies in its repeatability”, and that “it does not end with a single experience”, framing healing as a continual process that “provides health benefits and meaningful motivation in daily life”.

Collectively, these views present healing not as a one-time service, but as a long-term, structured practice embedded in daily life and community networks.

Compared to other types, Type 4 especially strongly agreed with the following statement:

- Although healing programmes can be expensive, they are worth the cost (Q30, Z = 1.492).

However, they most strongly disagreed with the following statement:

- To qualify as part of the healing industry, it must use natural environmental elements (Q12, Z = −1.245).

These results indicate that Type 4 participants emphasise the importance of sustaining long-term effects that carry over into everyday life through well-developed healing services.

Therefore, we labelled this type the “Sustainability-Oriented Type”.

## 4. Discussion

This study used Q methodology to explore how stakeholders—including policymakers, practitioners, and service users—perceive the healing industry. The analysis identified four distinct perception types: Type 1: Comprehensive and Service-Oriented, Type 2: Professionalism-Oriented, Type 3: Differentiation-Oriented, and Type 4: Sustainability-Oriented.

Participants in the Comprehensive and Service-Oriented Type (Type 1) believe the healing industry should function as a form of universal welfare accessible to the entire population. They emphasise an integrated healing approach that supports physical and mental well-being. Their perspective aligns with public choice theory’s conceptualisation of public goods—services characterized by non-excludability and non-rivalry in consumption [46]. Specifically, these participants view the healing industry as a public benefit that governments should provide to all citizens, not just to specific social groups. Their views reflect the traditional administrative state model, which prioritizes equity and universal access to public services.

Type 1 actively supports the healing industry as a core instrument for delivering public value. However, they demonstrated limited awareness of the need for critical assessment of policy effectiveness and accountability. When trust in policy precedes scrutiny, the objectivity and integrity of public service implementation may suffer.

This type consistently expressed a strong belief in the healing industry’s positive role, even while showing leniency toward ambiguous outcome measures or concerns about policy efficacy. They often adopted a supplier-oriented view and placed considerable trust in government-led service provision. This stance often reflected a belief in the government’s benevolence and pursuit of public interest rather than recognition of structural limitations such as the risk of government failure, an issue Buchanan [14] raised in public choice theory.

Public choice theory warns that bureaucratic self-interest and the influence of special interest groups can distort government action. Excessive trust in government-led initiatives without critical oversight may hinder efforts to verify service effectiveness and establish accountability [47]. According to Hallsworth et al. [48], civil servants and policymakers may overestimate the effectiveness of their policies and undervalue external feedback, particularly in the absence of institutional oversight. Such overconfidence bias, when coupled with weak monitoring structures, can lead to inefficiencies and a lack of transparency in public service delivery. This insight aligns with the core concerns addressed by behavioural economics and behavioural public choice theory, which emerged as theoretical complements to the limitations of traditional public choice theory.

Although Type 1 participants perceive the healing industry as a public good that should contribute to universal welfare, their perspective requires institutional mechanisms to gain policy legitimacy. Establishing a citizen–participatory performance evaluation system and a transparent feedback process, which links evaluation results directly to policy improvement, is essential. In this regard, Lee et al. [49] emphasised the need to create valuation frameworks based on social value and civic engagement to ensure more objective and legitimate evaluations of social policies. Type 1 participants would benefit from greater alignment with such evaluative practices.

The Professionalism-Oriented Type (Type 2) emphasises the need for structured operation and institutional development in the healing industry. They identified regulatory gaps, administrative fragmentation, and regional depopulation as key challenges requiring coordinated policy responses. Their perspective reflects a strong orientation toward industrial growth and systematic management, aligning with performance-based governance and service efficiency principles emphasised in public choice theory [15,50,51].

These participants advocated for establishing dedicated governmental bodies and clear operational guidelines. As policy consumers, they valued administrative clarity and predictability. They view the healing industry as a viable contributor to sustainable development, capable of building local industrial ecosystems, raising household incomes, and revitalising regional economies. To realize this potential, Type 2 participants stressed the importance of efficient resource allocation and performance-based accountability. They also highlighted the importance of role differentiation and professional expertise within the public sector to minimize inefficiencies and ensure responsible service delivery through coherent policy design.

Type 2’s emphasis on outcome-driven management, coupled with a continued commitment to public service delivery, aligns with public choice theory’s critique that government services often fail to reflect the public interest and risk inefficiency or shirking of responsibility [52,53]. Such awareness underscores the need for careful governance that recognises the limitations of public provision alone.

Consistent with this reasoning, Type 2 participants support expanding the roles of market mechanisms and civil society to accommodate the diverse societal demands and group preferences—an argument also advanced in public choice theory [53]. Their views align with the Organisation for Economic Co-operation and Development’s (OECD) policy recommendations that urge governments to balance public value with market efficiency [54,55,56]. The OECD suggests the government would act as a service provider, coordinator, and regulator. Accordingly, Type 2 participants call for policy designs that deliver strong performance outcomes while protecting equity and accessibility in public services [57].

Type 3 participants, the Differentiation-Oriented Type, view the healing industry critically. They argue that its concepts and terminology remain ambiguous and that healing services lack a clear distinction from existing wellness tourism or urban-based programmes. Their perspective of the healing industry is as a one-time experience that fails to provide lasting effects or convey sufficient uniqueness or value. Type 3 also raised concerns about the lack of public awareness and promotional efforts, which they believe contribute to low societal understanding and recognition.

These perspectives point to deeper issues of perception gaps and information asymmetry, as emphasised in public choice theory. Mann and Wüstemann [58] noted that in addition to reducing service delivery, information asymmetry creates mismatched expectations between providers and users, ultimately undermining institutional trust. From a behavioural public choice perspective, Viscusi and Gayer [13] warn that policy decisions made without accounting for cognitive biases and information deficits often result in poor design and misalignment with users’ actual needs. These insights help explain the conceptual confusion, institutional scepticism, and doubts about policy effectiveness voiced by Type 3 participants, who believe the public does not fully understand the healing industry.

Most participants in this type identified as service users and approached the healing industry from the standpoint of the citizen consumer. They tended to view healing services as a form of private consumption operating under the appearance of public provision. Accordingly, they prioritized access to transparent information and meaningful user engagement. Rather than passively accepting top-down policies, they call for policy evaluation and critique grounded in actual service experience. Their orientation aligns with a growing body of public choice literature that advocates for user-driven policy design and improved communication between institutions and the public [13,58,59].

Type 3’s critical stance stems from the healing industry’s vague conceptual boundaries, lack of differentiation, and insufficient dissemination of reliable information, all of which they see as contributing to growing policy distrust. To address these challenges, they call for clearer definitions and standardised terminology, the development of service quality standards, and a formal certification system to distinguish healing services from other offerings. Type 3 participants’ scepticism correlates with the industry’s information asymmetry, which we can more comprehensively explain by structural and economic factors, such as fragmented governance and the absence of standardised certification, which generate market confusion and erode trust in service credibility and quality [9,22,60]. From a health economics perspective, these issues reflect institutional inefficiencies that undermine the transparency, value, and equitable access of healing services [24]. Addressing such institutional shortcomings is critical to enhancing service differentiation and fostering broader public acceptance of the healing industry.

Lee et al. [9] point out that various ministries currently promote sector-specific healing programmes using inconsistent definitions and policy directions, highlighting the urgent need for an integrated conceptual framework. Accordingly, structural improvements to the economic system, the establishment of standardised certification mechanisms, and the policy design for clear service differentiation are emerging as practical solutions.

In light of these structural and economic challenges, Type 3 participants also emphasised the need for policies that deliver accurate information and incorporate user feedback mechanisms grounded in participatory governance. They argue that such features should reflect the expectations and lived experiences of citizen–consumers, thereby enhancing the legitimacy and responsiveness of public services. This position aligns with the work of Bovens and Zouridis [61], Christensen and Lægreid [62], and Viscusi and Gayer [13], who emphasise that reducing information asymmetries and bridging perception gaps requires user-centred policy design and inclusive evaluation processes.

Type 4 participants, the Sustainability-Oriented Type, view the healing industry as a life-integrated resource that produces long-term benefits for quality of life and well-being through repeated engagement. They emphasise that the essence of the healing industry lies in continuity and repeatability, and advocate institutionalising healing as a sustainable practice that supports daily life and provides personal meaning.

This group also stressed the importance of localizing operations, collaborating with community-based providers, implementing phased cooperation with experts, and ensuring consistent policy execution guided by clear operational principles. Their perspective reflects a critical awareness of democratic myopia—the tendency of policies to prioritize short-term gains over long-term outcomes—as discussed in public choice theory [63,64]. This type supports building governance structures capable of evaluating and upholding long-term policy sustainability. They recognize the value of healing programmes despite their costs and advocate for a rational balance between service quality and financial investment.

Type 4 participants also highlighted the importance of community-based participation structures. They emphasise that collaboration with professionals and repeated user involvement are essential for maximising the actual impact of healing policies. This position aligns with the concept of collaborative governance in public choice theory, which advocates coordinated cooperation between public institutions and private actors [65].

To implement such an approach, Type 4 participants propose institutionalising governance mechanisms that include central and local governments, private providers, residents, and multi-sectoral stakeholders (civil, public, industrial, and academic actors). They recommend creating systems for joint planning and execution, performance-based incentives, and user feedback loops that support iterative policy improvement. This governance model could help establish a more sustainable foundation for the healing industry while increasing public trust and acceptance.

The variation in perspectives across the four types revealed divergent policy expectations and interpretations among stakeholders. Rather than adopting a uniform policy model, the findings suggest the need for differentiated strategies that accommodate the multi-layered needs and values of diverse stakeholder groups. This complexity underscores the healing industry’s dual role as a convergent public service and market-oriented commodity.

In light of this duality, preference conflicts, information asymmetry, and trust gaps emerge as critical challenges—core concerns addressed in public choice theory. This observation highlights the need for multi-dimensional and adaptive policy frameworks. Such perception-based policy design can ultimately enhance national mental health outcomes by expanding nature-based healing services.

Building upon this, future policy design should not confine itself to differentiated service delivery and participatory evaluation systems; rather, it requires institutional arrangements that strengthen individual decision-making capacity and the adoption of scalable and sustainable financing mechanisms [66]. Such designs must explicitly address equity considerations across diverse population groups, along with the distribution of associated opportunity costs.

Distributional cost-effectiveness analysis (DCEA) provides a rigorous framework for evaluating trade-offs between equity and efficiency, thereby enabling policymakers to make evidence-informed, fair, and fiscally responsible decisions [67]. In this context, analytical perspectives drawn from public choice theory, behavioural economics, behavioural public choice theory, and health economics are essential for developing rational and scalable service delivery models, designing equitable resource allocation mechanisms, and establishing accountable governance systems in the healing industry through sustained empirical policy research.

## 5. Limitations and Future Research Directions

This study applied Q methodology to identify and interpret different stakeholder perception types regarding South Korea’s healing industry, applying public choice theory as its analytical lens. By identifying how various interest groups construct value, this study provides theoretical insights and practical implications—particularly given the healing industry’s dual role as a public good and an emerging sector. Nonetheless, several limitations inform directions for future research.

First, we used the strengths of Q methodology to explore stakeholders’ subjective perceptions in depth. Q methodology is well-suited for identifying individual perceptual structures and conducting comparative analyses, especially in conceptually ambiguous fields like the healing industry. However, it does not aim to produce generalisable typologies. To address this limitation, future research should adopt mixed-method designs that retain the Q methodology’s qualitative depth while incorporating quantitative surveys to examine the distribution of perception types and their correlations with demographic variables.

Second, this study focused on a specific set of stakeholders—namely, policymakers, practitioners, and service participants. It did not include the general public or potential users, who represent the primary beneficiaries of the healing industry. This exclusion limits the ability to capture broader social acceptance and demand-side perspectives. Future research should include a more diverse participant pool, particularly including members of the general public, to enable comparative analyses between service providers and service recipients.

Third, while we identified distinct perception types, we did not examine how these types influence actual policy processes and implementation outcomes. It remains unclear how perception types relate to variables such as policy acceptance, willingness to engage in institutional frameworks, or satisfaction with services. Future studies should investigate the behavioural implications of these perception types using quantitative methods. Comparative studies between provider-centred views and user experiences could also yield insights for improving the effectiveness and public legitimacy of healing industry policies.

In summary, this study used an exploratory approach to map the diverse perceptions of stakeholders surrounding the healing industry. It outlines key theoretical and policy implications tied to the multi-layered nature of policy design and the diversity of stakeholder demands. However, supporting the institutionalisation and long-term integration of the healing industry will require broader empirical investigation. Such efforts can help the industry evolve from a fragmented service model into a comprehensive public policy initiative that delivers public value and promotes long-term sustainability.

## 6. Conclusions

This study employed Q methodology within the framework of public choice theory to explore how various stakeholders perceive the healing industry. Through this approach, this study identified four distinct perception types, each reflecting different policy demands, expectations, and institutional challenges. These types revealed contrasting views on the industry’s target population, purpose, operational strategies, and sustainability. The findings highlight the importance of adopting differentiated policy approaches that address the complex and multi-layered demands of stakeholder groups rather than relying on a one-size-fits-all model.

This study particularly underscores that the healing industry functions as a public good and service commodity. Understanding the subjective perceptions of stakeholders is, therefore, critical to developing policies that are legitimate and effective.

Future policy design should address the following considerations:

First, to reconcile conflicting stakeholder preferences between universal accessibility and sustainable financing, it is essential to establish phased levels of coverage and clear prioritization criteria. Authorities should institutionalise a multi-stakeholder governance structure to ensure the involvement of diverse actors in the decision-making process.

Second, stakeholders should develop policy pathways to align the distinct needs identified across the four perception types with the payment structure of the broader health system. For instance, performance-based payment models or bundled payment schemes for nature-based healing services could simultaneously promote cost-efficiency and service quality.

Finally, a cost-effectiveness evaluation framework that quantitatively assesses the economic benefits and costs of the nature-based healing industry could enhance the sustainability of related policies.

These findings offer foundational insights for advancing the institutionalisation of the healing industry, designing governance frameworks, and establishing citizen–participatory evaluation systems. Based on these considerations, future empirical research should aim to enhance the health economic feasibility of such policies and support the development of policy designs that balance equity and efficiency.

## Figures and Tables

**Table 1:** Q methodology procedure.

Step	Procedure
Q concourse	399 literature research review collection
Q sample	39 statements extracted
P sample	25 participants selected
Q sorting	Forced distribution via www.qmethodsoftware.com
Data analysis	Ken Q Analysis (v1.3.1)

**Table 2:** Statistical significance of factors.

Content	Factor			
	1	2	3	4
Eigenvalue	9.249	2.879	2.022	1.379
Explained Variance (%)	37	12	8	6
Cumulative Variance (%)	37	49	57	63

**Table 3:** Correlations of the four types.

Factors	1	2	3	4
1	1.000			
2	0.4101	1.000		
3	−0.1451	0.1038	1.000	
4	0.5651	0.5512	−0.1912	1.000

**Table 4:** P sample and factor loading by type.

Type	P Sample	Factor Loading	Affiliated Sector(s)	Years of Experience
Type 1 N = 8	P3	10.000	Policy, External Collaboration	More than 11 years
	P15	5.754	Policy, Planning and Operations, Programme Delivery, External Collaboration, Expert, Other	6~10 years
	P13	4.493	Expert	6~10 years
	P21	4.346	Policy, Planning and Operations, Programme Delivery, External Collaboration, Expert	6~10 years
	P8	3.745	Policy, Planning and Operations, Programme Delivery, External Collaboration, Expert	6~10 years
	P5	3.425	Planning and Operations	Less than 1 year
	P10	2.354	Service User	N/A
	P7	1.859	Policy, Planning and Operations	Less than 1 year
Type 2 N = 8	P6	10.069	Planning and Operations	6~10 years
	P25	8.471	External Collaboration Expert	N/A
	P17	5.366	Service User	N/A
	P4	4.042	Programme Delivery, Other	Less than 1 year
	P2	3.780	Service User	N/A
	P16	3.585	Planning and Operations, Expert	More than 11 years
	P24	2.752	Policy, Planning and Operations, Programme Delivery, External Collaboration, Expert	More than 11 years
	P20	2.336	Policy, Planning and Operations, Programme Delivery, External Collaboration, Expert	6~10 years
Type 3 N = 3	P1	6.868	Service User	N/A
	P11	4.769	Service User	N/A
	P22	3.602	Service User	N/A
Type 4 N = 6	P9	6.304	Policy	Less than 1 year
	P12	3.233	Policy	1~3 years
	P18	2.951	Planning and Operations, Programme Delivery, External Collaboration	More than 11 years
	P23	2.835	Policy	1~3 years
	P14	2.081	Policy, Planning and Operations, External Collaboration	More than 11 years
	P19	1.724	Planning and Operations, External Collaboration	More than 11 years

## Data Availability

The datasets generated and/or analysed during this study are available from the corresponding author upon reasonable request.

## Supplementary Materials

The following supporting information can be downloaded at https://www.mdpi.com/article/10.3390/healthcare13161990/s1, Supplementary S1. Q statement, z-score and z-score values by type.
