# Supplementary material for: Stakeholders’ Perceptions of the Nature-Based Healing Industry in South Korea: A Q Methodology Study
Source: Healthcare (Basel). 2025 Aug 14;13(16):1990. doi: 10.3390/healthcare13161990 (PMC12386044; doi:10.3390/healthcare13161990)
Supplement: Supplementary file 1 [file healthcare-13-01990-s001.zip › healthcare-3711340-supplementary.pdf]

healthcare

MDPI

Supplementary S1. Q statement, z-score and z-score values by type.

No.	Statement	1		2		3		4	
		Z-Score	Q-Sort Value	Z-Score	Q-Sort Value	Z-Score	Q-Sort Value	Z-Score	Q-Sort Value
1	The healing industry should address physical and mental recovery in a balanced way.	1.59	4*	−1.02	−2	−0.19	−1	0.33	1
2	The healing industry must encompass social, cultural, and educational aspects of health.	1.31	3*	−0.54	−1	−0.14	0	0.58	1
3	The healing industry can even support spiritual recovery.	−0.95	−2	−1.21	−2	−1.14	−2	−1.08	−2
4	Healing programmes are more effective for physical health than for mental health.	−0.73	−1	−1.41	−4	−0.7	−2	−0.96	−2
5	The healing industry can be a part of complementary or integrative medicine.	0.81	1*	−0.72	−1	−0.59	−1	−0.2	−1
6	The healing industry is not about treatment but about maintaining health and improving quality of life.	1.3	2	−0.22	−1	−0.14	0	0.65	1
7	The healing industry helps form social bonds by providing opportunities for interaction.	1.05	2	−0.06	0	−1.4	−3*	0.44	1
8	Healing services should be made available to the entire population.	1.67	4*	−0.07	0	−0.6	−2	0.19	0
9	The healing industry should exclude individuals with medical conditions requiring treatment.	−1.78	−4	−1.4	−3	−0.21	−1*	−2.27	−4
10	The perception of the healing industry is as a form of welfare that supports vulnerable or marginalized groups.	−0.26	0*	−1.19	−2	−1.24	−3	−1.09	−2
11	The intended users of the healing industry remain unclear.	−1.62	−4	−1.28	−2	0.42	1*	−0.91	−2

No.	Statement	1		2		3		4	
		Z-Score	Q-Sort Value	Z-Score	Q-Sort Value	Z-Score	Q-Sort Value	Z-Score	Q-Sort Value
12	To qualify as part of the healing industry, it must use natural environmental elements.	0.68	1	0.14	0	−0.11	0	−1.25	−3*
13	Experiencing a connection with nature is important in the healing industry.	0.88	2	0.09	0	−0.38	−1	0.29	0
14	Scientifically and medically proving the effectiveness of the healing industry can help with its promotion.	1.49	3	0.48	1*	−0.74	−2*	1.33	3
15	The healing industry requires a redefinition of concepts and terminology with clear boundaries.	0.09	0	0.21	0	1.03	2	1.39	3
16	Healing programmes often feel indistinguishable from general wellness tourism.	−1.52	−3	−1.32	−3	1.7	4*	−1.78	−4
17	Cross-sectoral cooperation between policy, health, psychology, and medical fields is necessary to advance the healing industry.	1.44	3	0.57	1	0.93	2	0.83	2
18	The healing industry needs a government body dedicated to its oversight.	−0.65	−1	1.89	4*	1.02	2*	−0.87	−1
19	The healing industry can serve as a future-oriented strategy to address rural depopulation.	0.69	1	1.8	3*	0.26	0	0.87	2
20	A business structure that ensures income for residents is crucial for the sustainability of the healing industry.	0.07	0*	1.19	2	1	2	0.95	2
21	Despite initial attention and investment, there is insufficient maintenance and repair of healing facilities.	0.05	0	0.9	2	0.72	1	−0.79	−1*
22	The healing industry needs clear and systematic guidelines for its operation and management.	0.84	2	1.8	4	1.28	3	−0.12	0*
23	Due to a lack of public promotion, general awareness of healing programmes remains low.	−0.55	−1*	0.62	1	1.19	3	0.51	1

No.	Statement	1		2		3		4	
		Z-Score	Q-Sort Value	Z-Score	Q-Sort Value	Z-Score	Q-Sort Value	Z-Score	Q-Sort Value
24	Collaboration with experts in the planning and operation phases is essential for administrative practitioners.	0.44	1	1.3	3	−0.18	0	1.6	4
25	National economic development should be the ultimate policy goal of the healing industry.	−0.44	−1	0.63	2*	−1.07	−2	−0.36	−1
26	Formal facility standards overly constrain the healing industry.	−1.19	−2	−1.55	−4	0.15	0	0.06	0
27	There is a need to integrate healing approaches across fields such as forestry, agriculture, marine, and gardening.	0.44	1	0.08	0	−0.58	−1	1.17	2*
28	The success of the healing industry depends on the participation and cooperation of the local community.	0.63	1	0.74	2	0.79	1	0.82	2
29	Korea's healing industry has developed to the point of attracting global attention.	0.06	0	0.13	0	−2.23	−4*	0.17	0
30	Although healing programmes can be expensive, they are worth the cost.	−0.99	−2*	0.45	1*	−2	−4*	1.49	3*
31	There are few clearly recognized success cases in the healing industry.	−0.42	−1	−0.78	−1	0.99	2*	−0.13	−1
32	Public-private partnerships, including universities, can enhance the sustainability of the healing industry.	0.24	0	0.84	2	0.42	1	0	0
33	The healing industry should pursue long-term effects through repeated and continuous experiences rather than one-time programmes.	0.83	2	1.29	3	−0.49	−1*	1.68	4
34	In some cases, the development of healing infrastructure and commercial interests end up damaging nature.	−1.47	−3	−1.38	−3	−0.18	0	−1.01	−2
35	The healing industry contributes to raising environmental awareness.	−0.37	−1	0.52	1	−1.75	−3*	0.2	0
36	The effects of healing programmes often fade quickly after the experience ends.	−1.11	−2	−1.07	−2	1.21	3*	−1.51	−3

No.	Statement	1		2		3		4	
		Z-Score	Q-Sort Value	Z-Score	Q-Sort Value	Z-Score	Q-Sort Value	Z-Score	Q-Sort Value
37	Indoor healing programmes often lack differentiation from those offered in urban settings.	−0.3	0	−0.78	−1	1.59	4*	−1.34	−3
38	Researchers have not clearly defined how they are measuring the outcomes of the healing industry.	−1.31	−3*	−0.27	−1	0.79	1	0.38	1
39	Success cases from other countries often do not fit Korea's context and require adaptation.	−0.91	−2	0.61	1	0.57	1	−0.25	−1

Note. *$p < 0.05$
